# Internet Search Activity of Young People With Mood Disorders Who Are Hospitalized for Suicidal Thoughts and Behaviors: Qualitative Study of Google Search Activity

**DOI:** 10.2196/28262

**Published:** 2021-10-22

**Authors:** Khatiya C Moon, Anna R Van Meter, Michael A Kirschenbaum, Asra Ali, John M Kane, Michael L Birnbaum

**Affiliations:** 1 Department of Psychiatry Zucker Hillside Hospital Glen Oaks, NY United States; 2 Department of Psychiatry The Donald and Barbara Zucker School of Medicine at Hofstra/Northwell Hempstead, NY United States; 3 Feinstein Institute of Medical Research Manhasset, NY United States

**Keywords:** suicide, mood disorders, depression, internet, search engine, Google search, digital health, mobile health, adolescent, young adult

## Abstract

**Background:**

Little is known about the internet search activity of people with suicidal thoughts and behaviors (STBs). This data source has the potential to inform both clinical and public health efforts, such as suicide risk assessment and prevention.

**Objective:**

We aimed to evaluate the internet search activity of suicidal young people to find evidence of suicidal ideation and behavioral health–related content.

**Methods:**

Individuals aged between 15 and 30 years (N=43) with mood disorders who were hospitalized for STBs provided access to their internet search history. Searches that were conducted in the 3-month period prior to hospitalization were extracted and manually evaluated for search themes related to suicide and behavioral health.

**Results:**

A majority (27/43, 63%) of participants conducted suicide-related searches. Participants searched for information that exactly matched their planned or chosen method of attempting suicide in 21% (9/43) of cases. Suicide-related search queries also included unusual suicide methods and references to suicide in popular culture. A majority of participants (33/43, 77%) had queries related to help-seeking themes, including how to find inpatient and outpatient behavioral health care. Queries related to mood and anxiety symptoms were found among 44% (19/43) of participants and included references to panic disorder, the inability to focus, feelings of loneliness, and despair. Queries related to substance use were found among 44% (19/43) of participants. Queries related to traumatic experiences were present among 33% (14/43) of participants. Few participants conducted searches for crisis hotlines (n=3).

**Conclusions:**

Individuals search the internet for information related to suicide prior to hospitalization for STBs. The improved understanding of the search activity of suicidal people could inform outreach, assessment, and intervention strategies for people at risk. Access to search data may also benefit the ongoing care of suicidal patients.

## Introduction

The high prevalence of suicidal behaviors is a public health crisis. Suicide is a leading cause of death among young people in the United States and accounts for nearly 1 million deaths annually worldwide [1]. Suicidality is a major target of both preventative public health efforts and clinical behavioral medicine. Nonetheless, after decades of research, little progress has been made in the prediction and reduction of suicide incidence [2]. Innovative approaches are needed to identify individuals at high risk for suicide and to engage them in care.

Technology has the great potential to aid these efforts by improving the current methods for assessing suicidal thoughts and behaviors (STBs) both in and outside of clinical settings. STBs are traditionally evaluated with intermittent semistructured patient interviews or with patient self-report measures. Unfortunately, both purposeful concealment and the underreporting of STBs are common [3,4]. Additionally, traditional clinical assessment does not occur often enough to reveal the day-to-day fluctuations of STBs that patients experience [5-7]. Prior studies have demonstrated that data gathered from various digital platforms, including smartphone apps, wearable devices, social media, and internet search engines, can enhance the traditional evaluation of STBs and other psychiatric symptoms [5,6,8-10]. The use of these information sources is acceptable to patients [9,11,12], and data gathered from digital platforms can be collected passively in real time and in the same settings in which patients are likely to experience symptoms. Thus, digital data have greater potential to provide a more complete assessment of STBs compared to data collected via traditional methods. There is also emerging evidence that aggregates of digital data can be used to create digital phenotypes—clusters of symptoms and behaviors that correlate with outcomes—of patients with STBs [13]. The improved characterization of these phenotypes could enhance suicide risk prediction models for both individuals and populations.

Little is known about the internet search activity of people with STBs, but this digital data source has the potential to improve both clinical and public health efforts, such as suicide risk assessment and prevention. Google is the most highly trafficked website in the world [14]. In a survey, a majority (59%) of young people reported learning about suicide from web-based sources [15]. Young people who report that they engage in STBs also report that they have been exposed to web-based, suicide-related content [16,17]. Incorporating search data into the assessment of STBs confers a number of advantages over traditional clinical assessment methods. For example, search data do not rely on self-reports, making these data less susceptible to purposeful concealment, accidental omission, or recall bias. Additionally, search data leave a longitudinal record that can provide a more accurate characterization of the temporal evolution of STBs. Finally, search data can facilitate the delivery of search engine–driven, just-in-time interventions that aim to prevent suicide attempts—a feat that is incredibly challenging to accomplish with the current interval clinical assessment model [10].

Most previous literature on examining suicide-related internet search activity has relied on either population-level data from search trends or self-reports of search activity from samples of participants in the community whose suicide risk levels and psychiatric histories are unknown. These methods have some important limitations, including the fact that population-level data do not necessarily correlate with individual-level behavior patterns, thus limiting their clinical utility [18]. Additionally, studies that rely on self-reports are limited by recall bias as well as a lack of objective corroboration of symptoms and behaviors.

To determine if search data are capable of providing information on the clinically meaningful contexts surrounding suicidality, we performed a qualitative analysis of search data collected from adolescents and young adults who were diagnosed with mood disorders and hospitalized for STBs. To our knowledge, this is the first study in which search archives were extracted directly from participants with confirmed clinical diagnoses and established STBs. We hypothesized that participants’ search activity would include references to behavioral health and suicide-related information in the months before a psychiatric hospitalization for STBs.

## Methods

### Recruitment

Participants aged between 15 and 30 years who had previously been diagnosed with a primary mood disorder were screened for eligibility by Northwell Health’s Zucker Hillside Hospital inpatient and outpatient psychiatric departments. We focused on individuals with mood disorders because STBs are a more common reason for hospitalization in this population. Only individuals who had been hospitalized due to STBs were included in this study. Recruitment occurred between March 2016 and December 2018. This study was approved by the Institutional Review Board of Northwell Health. Written informed consent was obtained from adult participants and legal guardians of participants aged under 18 years. Assent was obtained from participating minors. All participants were receiving treatment as usual.

### Data Collection

Participants were asked to extract their search activity by logging on to their Google account and requesting their data archive. Participation involved 1 to 2 visits after consent, during which all historical search data were requested, downloaded, and collected. These data archives included user-generated search terms that were time-stamped. Historical clinical data, including dates of psychiatric hospitalizations, presenting symptoms, and diagnoses, were obtained from medical records.

For this study, we focused just on search data from the 3-month period prior to each psychiatric hospitalization for STBs. We selected 3 months because we thought that this amount of time represented a period that was long enough to adequately capture suicide-related searches (SRSs) that were conducted as symptoms escalated to the point of necessitating hospitalization. If a participant experienced multiple hospitalizations, each hospitalization was evaluated as a distinct entity with its own corresponding search history. Thus, there were more search histories than there were participants. If a participant had multiple hospitalizations in a 3-month period, overlapping search queries were only analyzed once.

### Data Analysis

Five reviewers (authors AA, MLB, MAK, ARVM, and KCM) simultaneously manually reviewed each search query and extracted those that included terms related to suicide and behavioral health. Relevant searches were highlighted. The findings were then discussed as a team to reach a consensus and identify relevant search categories. Extracted search terms were then coded into 5 thematic categories. These included STBs, help seeking, symptoms of mental health disorders, trauma and negative life events, and drugs of abuse. A similar method of categorization was previously used successfully by our group in a study of the search queries of patients with psychosis [8]. These thematic categories coincided with factors that are recognized as modulators of suicide risk [19] and were chosen because they were thought to be sufficiently broad enough to capture a wide array of behavioral health–related symptoms and behaviors that are searched for by individuals.

The reviewers excluded ambiguous searches unless additional clinical context was available. For example, searches involving the name of prescription medications that could also be used as drugs of abuse (ie, Adderall [dextroamphetamine-amphetamine] and Xanax [alprazolam]) were counted in the analysis only if they included additional content that could guide categorization (eg, “how to overdose on klonopin” or “side effects of adderall”) but not if they were limited to the name of the medication alone. We also excluded searches that were not germane to suicide or behavioral health.

The reviewers familiarized themselves with the available clinical documentation prior to the assessment of search histories. The available clinical documentation included patient demographics, the primary diagnosis, the reason for hospitalization, and the attempted or planned method of suicide. Knowledge of the clinical documentation was used to contextualize search content and assess if flagged search queries were in fact related to the participants’ clinically documented symptoms and behaviors. Reviewers met as a team to resolve discrepancies in the appropriate categorization of individual search queries.

## Results

### Participants’ Characteristics and Search Themes

A total of 43 participants provided access to their Google search data. These 43 participants had 63 hospitalizations for STBs (number of hospitalizations per person: mean 1.5). Search histories from the 3-month period prior to each hospitalization were extracted and analyzed. A total of 37,738 search queries were reviewed. Participant demographics are summarized in Table 1. The prevalence of different search themes among participants is presented in Table 2.

**Table 1:** Participant demographics.

Characteristic		Value
Age (years), mean (SD)		20.6 (3.1)
**Gender, n (%)**		
	Male	18 (42)
	Female	25 (58)
**Race, n (%)**		
	Black or African American	8 (19)
	Asian American or Pacific Islander	6 (14)
	White	21 (48)
	Mixed or other	8 (19)
**Ethnicity, n (%)**		
	Hispanic or Latino	10 (23)
	Not Hispanic or Latino	32 (74)
	Unknown	1 (2)
**Primary diagnosis, n (%)**		
	Major depressive disorder	37 (86)
	Bipolar disorder	5 (12)
	Unspecified mood disorder or other	1 (2)

**Table 2:** Prevalence of search themes.

Search theme	Number of participants (%)	Number of search histories^a^ (%)
Suicide	27 (63)	36 (57)
Help seeking	33 (77)	43 (68)
Substance use	19 (44)	25 (40)
Mood and anxiety symptoms	19 (44)	24 (38)
Trauma and negative life events	14 (33)	17 (27)

^a^Each search history contains searches that were conducted during the 3-month period prior to a unique hospitalization.

### SRS Queries

Queries related to suicide were found in 36 search histories representing 27 out of the 43 (63%) unique participants. Participants’ SRSs varied in terms of their temporal proximity to hospitalization. Further, 4 participants conducted all of their SRSs in the week preceding hospitalization, 6 participants conducted all of their SRSs within 3 weeks before hospitalization, and 14 participants conducted all of their SRSs ≥4 weeks before hospitalization.

Exemplars of search queries are presented in Table 3. SRSs included queries related to the television show *13 Reasons Why*—a popular show about a teenage girl who dies by suicide that was airing during the recruitment period—as well as other suicide-related topics in news media. Some participants searched for highly specific and unusual suicide methods. For example, one participant conducted numerous searches related to giving themselves cancer and was investigating the possibility of purchasing live cancer cells on web-based platforms. This participant also searched for a specific species of poisonous frog and investigated the cyanide content of apple seeds. Another participant performed numerous searches related to methods, such as how to overdose on various medications, “how to take apart a shaving razor,” and “how to get the blade out of a pencil sharpener.” Another participant searched “easy ways to get pneumonia,” and another searched “how long can a human go without food.” Other participants conducted SRSs that were less specific but nonetheless indicated thoughts about death. For example, one simply searched “I want to die,” another queried “would you say suicide is a cry for help,” and a third asked “why wont I just kill myself already?” Search queries about suicide-related tattoos, famous suicide-related quotes, and literature about suicide were made by 6 participants. Searches for suicide chat rooms were conducted by 2 participants.

Of the 43 participants, 9 (21%) searched for information that was directly related to their planned or chosen method of suicide. These 9 participants were represented by 10 search histories. In all of these cases, the planned or used method of suicide was drug overdose, as noted in the clinical record. For example, one such participant who was hospitalized after attempting suicide by overdosing on NyQuil (doxylamine and acetaminophen) conducted 8 searches in the week before hospitalization regarding overdosing on NyQuil, including “how many Nyquil [sic] pills does it take to kill me?”

**Table 3:** Notable search queries.

Themes and participants		Queries
**Suicide**		
	Participant A	“pro suicide chat rooms”
	Participant B	“giving your self cancer” “live cancer cells for sale” “poison dart frog for sale” “apple seeds cyanide”
	Participant C	“how to take apart a shaving razor” “how to get the blade out of a pencil sharpener” “how painful is slitting your wrists” “if you bang your head against the wall can you die” “least painful way to commit suicide”
	Participant D	“best pills to overdose on”
	Participant E	“would you say suicide is a cry for help”
**Help-seeking behavior**		
	Participant A	“mobile crisis hotline” “psychiatrist works with aetna” “top psychiatrist near me”
	Participant B	“how does a therapist help with anxiety”
	Participant C	“how does a psychologist decide if you need medication” “what does a psychologist do”
	Participant D	“can I turn myself into a mental hospital”
**Drugs and other substances**		
	Participant A	“funniest things to do after eating an edible” “does heating up weed make it smell”
	Participant B	“cbd for newbies”
**Mood and anxiety symptoms**		
	Participant A	“do you have to cut yourself to be depressed” “what make me doubt myself all the time” “what does dreading everything mean” “why are there days where I be confused and don’t know what to do and I have a feeling in my chest” “what if I don’t know what I want to do for my future because I don’t see a future for myself” “waking up with a panic feeling in my chest”
	Participant B	“everything seems too overwhelming and pointless”
	Participant C	“screaming in sleep” “different person after depression”
	Participant D	“how to be happy” “I am so lonely”
	Participant E	“I am so done”
**Trauma and negative life events**		
	Participant A	“how do you get over a heartbreak?”
	Participant B	“how to deal with losing a best friend”

### Help-Seeking Searches

Queries related to behavioral health care were found in 43 search histories representing 33 of the 43 participants (77%). Searches regarding suicide hotlines or crisis lines were only conducted by 3 participants. Most commonly (n=13), search histories contained queries regarding outpatient resources, including searches for specific behavioral health clinics and general information about outpatient behavioral health care. The names of specific behavioral health providers were queried 11 times. Information about the etiology of, differences among, and clinical course of different psychiatric disorders were queried in 12 search histories. Queries regarding inpatient psychiatric care were found in 10 search histories, including questions about the conditions and rules of a psychiatric unit. For example, one participant searched whether they would be able to use their phone while hospitalized. Search histories also contained queries about substance abuse resources (n=4), crisis resources (n=4), public assistance programs (n=2), and social support (n=3). In 5 search histories, participants searched for alternative interventions, such as pet therapy, meditation apps, and complementary medicine. In 19 search histories, 15 of the 43 participants (35%) searched for information about psychiatric medications, including side effects, desired effects, dosages, and general psychoeducation.

### Drugs and Other Substances

Queries related to drugs of abuse were found in 25 search histories representing 19 of the 43 (44%) participants. Alcohol was the most commonly queried substance; it was represented in 13 search histories. Cannabis-related searches were present in 10 search histories. Two participants searched for unusual ways to attain intoxication; one asked “can ibuprofen get you high,” and the other queried “how to properly get high off computer duster.” One participant conducted a search about purchasing a drug testing kit. Other participants conducted searches for salvia, ketamine, tobacco and nicotine, amphetamines, and crack cocaine.

### Mood and Anxiety Symptoms

Queries related to mood and anxiety symptoms were found in 24 search histories representing 19 of the 43 individuals (44%). Such searches included those for music, movies, tattoos, internet memes, and quotes related to depression. For example, one participant searched “depression movies,” another searched “mental illness quotes” and “tattoos about depression,” and another searched “15 saddest country songs.” Other participants searched for information related to despair. For example, one participant queried “I am so lonely,” another queried “can no longer focus on anything or learn,” and a third queried “everything seems too overwhelming and pointless.”

### Trauma and Negative Life Events

Queries related to traumatic or otherwise negative life events were found in 17 search histories representing 14 of the 43 participants (33%). In this category, 4 participants conducted searches related to sex crimes, including searches about how to report rape, how to escape domestic violence, and general information regarding sexual assault and harassment and searches related to support groups for sexual assault survivors. Further, 3 participants conducted searches related to unwanted pregnancies, including those about seeking abortion and emergency contraception. Additionally, 7 participants conducted searches related to difficulties in interpersonal relationships, including the dissolution of friendships and romantic relationships. Finally, 1 participant searched for information on dealing with bullies, and 2 participants conducted searches related to notorious gun violence events, including the Newtown, Connecticut, school shooting.

## Discussion

### Principal Findings

In this study we explored the internet search queries of young people with mood disorders that were made in the months leading up to a hospitalization for STBs. This study yielded a number of important findings. Our study confirms that patients with STBs conduct SRSs and other clinically meaningful searches prior to hospitalization.

A majority (27/43, 63%) of the participants in our sample conducted SRSs. This finding is consistent with those of previous research showing that people with self-reported STBs search for suicide-related content on web-based platforms [20,21]. However, to our knowledge, ours is the first study to extract search data from individuals with clinically confirmed STBs. Additionally, in a sizable minority of cases (9/43, 21%), suicide methods that were searched exactly matched participants’ planned or selected method of attempting suicide in real life. Although more research is needed to understand how the presence or content of SRSs relates to suicidal behavior, our data suggest that SRSs may indicate a heightened suicide risk because, at least for some individuals, the internet likely aids suicide planning.

Search queries that were not explicitly related to suicide nonetheless revealed highly sensitive and clinically meaningful experiences that can otherwise go unreported in a traditional clinical interview. For example, searches about experiences with sexual abuse, the dissolution of relationships, bullying, loneliness, panic, and substance use could put clinical symptoms into context if they are known to a clinician. In addition, many searches related to the self-expression of mood symptoms were found in our sample, such as searches regarding movies, books, memes, and tattoos about depression. This finding suggests that suicidal young people are interested in consuming and sharing content related to their experiences with behavioral health, though the influence that this type of content has on symptoms and behaviors is unclear.

We also found that a majority (33/43, 77%) of our participants searched for help seeking–related information, including information about behavioral health services and psychoeducational resources. Unfortunately, there is a substantial body of prosuicide content on the internet and evidence that exposure to prosuicide content can increase the incidence of suicidal ideation and worsen mood symptoms in young people [22,23]. Our data reinforce the need for point-of-search interventions that counteract prosuicide information. The fact that young people are searching for help-seeking and suicide-related information on web-based platforms represents a potential opportunity to intervene. For example, if help-seeking searches were known to a clinician, they could serve as a jumping-off point for talking about treatment options. In addition, although search engines currently return information for suicide prevention hotlines when people search for the term *suicide*, we have shown that many suicide-related, mood-related, and help-seeking searches do not include this term. Only 3 individuals in our sample specifically searched for suicide hotlines, suggesting that this may not be an appealing intervention for many individuals. Optimizing algorithms to identify likely suicide-related, symptom-related, and help-seeking terms may result in the development of more impactful point-of-search interventions and improve pathways to care.

Previous research supports the idea that the incidence of suicidal ideation does not increase in a linear fashion in the days immediately preceding a suicide attempt and has a high level of moment-to-moment variability over a given period of time [6,7,24]. Such research also suggests that individuals with STBs can be grouped into different phenotypes—those whose suicidal ideation varies considerably in frequency and intensity and those whose suicidal ideation is more stable over time. These different groups may require different types of assessment and intervention. In our sample, 4 participants completed all of their SRSs in the week before hospitalization, possibly suggesting that their STBs were escalating prior to hospitalization. In contrast, 14 individuals stopped conducting SRSs 3 weeks before their hospitalization, possibly suggesting that the STBs that resulted in hospitalization may have been more impulsive. Although additional research is required to understand the motivation behind and impact of each search, our findings support the notion that different patterns in the temporal evolution of SRSs exist and suggest that internet search data, along with other digital and clinical data, can be used to identify STB phenotypes and tailor interventions accordingly in the future [13].

### Limitations

Our study has several important limitations. First, the study was conducted at a single site with a small, albeit diverse, population of young people aged 15 to 30 years. The generalizability of our results is therefore limited. In addition, we are unable to comment on the prevalence or content of SRSs among older adults or younger children. This study was limited to participants with mood disorders; comorbidities and other primary diagnoses were not considered.

Second, our study lacked a formal comparison group, so we cannot state conclusively that the results are unique to individuals with STBs. However, notably, a similar previous study of individuals with psychosis did not yield significant numbers of SRSs, suggesting that these types of searches do not occur at similar rates across patient populations [8].

Third, search queries alone do not provide enough context for confidently stating the motivations and intentions behind each search. For example, searches about suicide chat rooms or memes could represent suicidal intent, help-seeking behavior, curiosity, or other intentions. It is also possible that people with access to participants’ devices conducted searches while on participants’ accounts, further limiting our understanding of participants’ intentions. We sought to mitigate uncertainty by cross-referencing search queries with clinical data when such data were available in the medical records, which provided context for categorization, and conferring with one another to resolve ambiguity in the meanings of or intentions behind queries. However, even these actions introduce bias into the assessment and thematic categorization of search queries. Future studies should consider accessing browser histories, which could provide additional information that is related to motivation and is based on what websites participants visited after making their queries.

Fourth, it is possible that some clinically relevant search queries were not captured in our data set. Participants who were motivated to conceal their searches may have done so. For example, search queries that were conducted while participants used Google’s incognito mode were unavailable to us, as were searches that participants deleted prior to the download of this study’s data. In addition, searches that were conducted through alternative search engines were not assessed in this study.

Finally, we only analyzed searches that were conducted 3 months before a hospitalization. It is possible that individuals conduct SRSs well before this time or that only searches that are conducted in close temporal proximity to hospitalization have predictive value. Future research should consider not only the temporal association between an individual’s searches and STBs but also whether the time of day, search frequency, or other non–content-related information about searches relates to suicide risk.

### Future Directions

The understanding that people at high risk for suicide are indeed searching for suicide-related content represents an opportunity to use these data for treatment planning and risk assessment. For example, in the future, internet search data could potentially be used as clinical collateral, particularly during initial psychiatric assessments where patients may be hesitant to share their experiences with STBs. Additionally, the search data of established patients could augment infrequent psychiatric assessments by allowing for the closer monitoring of STBs in between clinical appointments. Access to this information can help both clinicians and patients understand STBs in the context of surrounding life events and mood symptoms with more granularity than is currently typical.

Although it is currently not possible to systematically use search data in clinical evaluation and management, patients can download and share their own web-based activity with their treatment providers. However, for this to occur, statistical tools that can process search data and return relevant information in a format that is clinically usable and scalable need to be developed. Additionally, our work suggests that search histories can be used to establish novel risk factors for suicide. Ultimately, the further clarification of such risk factors may be better suited to more robust statistical approaches (such as machine learning approaches) for analyzing larger numbers of participants. Furthermore, questions about legal considerations, effectiveness, integration into clinical workflows, and reimbursement also need to be addressed before these methods can reasonably be implemented.

Finally, the clinical use of internet search data must be undertaken with the utmost concern for patient privacy. Search data often contain highly sensitive information that people may be uncomfortable sharing. If search data are to be integrated into clinical workflows or search engine–led interventions, it is important that users consent to the review of search data and that they be continuously informed about how these data might be used in a transparent and collaborative manner.

## Abbreviations

SRS: suicide-related search
STB: suicidal thought and behavior
